# Mending a broken heart: current strategies and limitations of cell-based therapy

**DOI:** 10.1186/s13287-020-01648-0

**Published:** 2020-03-26

**Authors:** Lee Chuen Liew, Beatrice Xuan Ho, Boon-Seng Soh

**Affiliations:** 1 Disease Modeling and Therapeutics Laboratory, A*STAR Institute of Molecular and Cell Biology, 61 Biopolis Drive Proteos, Singapore, 138673 Singapore; 2 Department of Biological Sciences, National University of Singapore, Singapore, 117543 Singapore; 3 Key Laboratory for Major Obstetric Diseases of Guangdong Province, The Third Affiliated Hospital of Guangzhou Medical University, Guangzhou, 510150 China

**Keywords:** Ischemic heart disease, Cell-based therapy, Pluripotent stem cells, Cardiomyocytes, Exosome

## Abstract

The versatility of pluripotent stem cells, attributable to their unlimited self-renewal capacity and plasticity, has sparked a considerable interest for potential application in regenerative medicine. Over the past decade, the concept of replenishing the lost cardiomyocytes, the crux of the matter in ischemic heart disease, with pluripotent stem cell-derived cardiomyocytes (PSC-CM) has been validated with promising pre-clinical results. Nevertheless, clinical translation was hemmed in by limitations such as immature cardiac properties, long-term engraftment, graft-associated arrhythmias, immunogenicity, and risk of tumorigenicity. The continuous progress of stem cell-based cardiac therapy, incorporated with tissue engineering strategies and delivery of cardio-protective exosomes, provides an optimistic outlook on the development of curative treatment for heart failure. This review provides an overview and current status of stem cell-based therapy for heart regeneration, with particular focus on the use of PSC-CM. In addition, we also highlight the associated challenges in clinical application and discuss the potential strategies in developing successful cardiac-regenerative therapy.

## Introduction

Ischemic heart disease, the most common type of cardiovascular disease, is characterized by the restriction of blood flow to the heart muscle that results from the plaque build-up in the arteries [1]. This obstruction of coronary arteries limits the oxygen supply to the heart tissue and thus causes irreversible death of cardiomyocytes (myocardial infarction). As the disease progresses, especially if left untreated, the expansion of the infarcted zone will impair the function of the left ventricle, which would eventually lead to heart failure [2]. The current therapeutic options available, including pharmaceutical drugs, such as angiotensin-converting enzyme inhibitors, beta-blockers, diuretics, and angiotensin receptors blockers, are mainly symptomatic treatments that could merely alleviate the manifestation of the disease. Similarly, although cardiac revascularization therapy with percutaneous coronary intervention (PCI) and coronary artery bypass grafting (CABG), as well as the use of cardiac implantable electronic devices could improve the patient survival and quality of life, they have been ineffective in preventing the disease progression [3, 4]. While heart transplantation is thought to be the only curative treatment for patients with end-stage heart failure, the shortage of organ donors that limit the number of possible heart transplants and the associated post transplant complications makes this treatment inapplicable as yet.

**Fig. 1:**
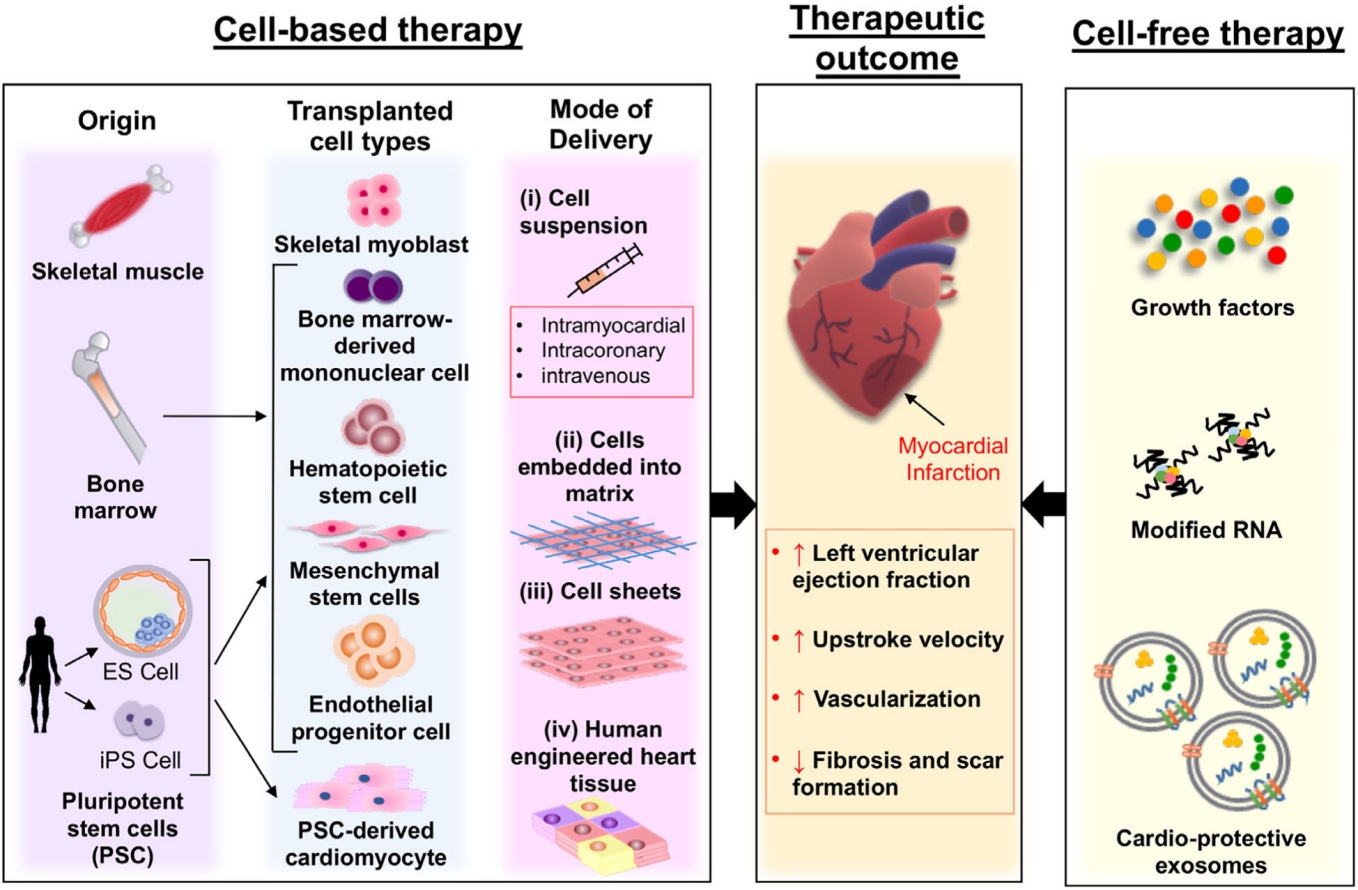
Current strategies for successful cardiac regenerative therapy

Cardiovascular research has largely been focused on cell-based therapy for myocardial repair, with particular emphasis on replacement and/or restoration of the damaged myocardium [5, 6]. For the past two decades, immense efforts have been put into experimenting a myriad of transplantable cell types for their use in cardiac regeneration. These include skeletal myoblasts, bone marrow mononuclear cells (BM-MNC), hematopoietic stem cells (HSC), endothelial progenitor cells (EPC), mesenchymal stem cells (MSC), and pluripotent stem cell (PSC)-derived cardiomyocytes (CM) (PSC-CM) (Fig. 1). While seemingly favorable outcomes do come into view, no one ideal cell type has yet to emerge. This review aims to evaluate the existing state of cell-based therapy for heart disease, with a particular focus on PSC-CM. Herein, we also discuss the current translational challenges of PSC-CM and their future perspectives for successful cardiac regenerative therapy.

## Cell-based therapy

Cardiomyocytes are terminally differentiated cells that lack regeneration potential, thereby limiting their ability to restore the cardiac function after serious injury [7, 8]. In order to repair the myocardium, the most promising approach is to remuscularize the damaged tissue by replacing the lost cardiomyocytes, which could be done by transplantation of cells that are capable of cardiomyogenesis. Consequently, the search for the most suitable cell type for transplantation is therefore doubtlessly a crucial task. Table 1 summarized the cell types used for cardiac therapy and regeneration in pre-clinical and clinical studies.

**Table 1:** Summary of different cell types and animal models used in cardiac repair and regeneration

Cell types		Model	Outcome	Mode of delivery	References
Pluripotent stem cells	Human-induced pluripotent stem cell-derived cardiomyocytes (iPSC-CM)	Rat	Improved cardiac function and neovascularization	Cell sheet transplantation at myocardium	[9]
		Pig	Increased ejection fraction and decreased myocardial wall stress	Cell sheet transplantation at epicardial	[10]
	Human embryonic stem cell-derived cardiomyocytes (hESC-CM)	Rat	Improved cardiac function	Injection in left ventricular wall	[11]
		Pig	Improved cardiac function	Embryoid bodies transplantation at posterolateral wall of the left ventricle	[12]
		Non-human primates	Remuscularization of infarcted zone	Intramyocardial injection	[13]
		Mouse	Improved cardiac function	Injected beneath the coronary artery ligation	[14]
	Human embryonic stem cell-derived cardiovascular progenitors	Human	Most patients were symptomatically improved with an increased systolic motion of the cell-treated segments	Sub-epicardial injection of cells in a fibrin patch	[15]
Adult stem cells	Bone marrow-derived mononuclear cells (BM-MNC) and progenitor cells	Human	Improved LVEF	Intracoronary infusion	[16–18]
			No improvement of LVEF		[19]
	Bone marrow-derived mesenchymal stem cells (BM-MSC)	Human	Reduced infarct size and scar formation	Transendocardial injection	[20, 21]
		Rat	Increased LVEF	Epicardial placement of a MSC-sheet	[22]
	Skeletal muscle-derived stem cells	Human	Improved LVEF	Sub-epicardial injection	[23]

### Adult stem cells

Adult stem cells would probably be the first option that comes into one’s mind, owing to their ability to develop into all cell types of the organ from which they originate and steer clear of the controversial ethical issue that has been haunting the use of embryonic stem cells. Skeletal myoblast, a precursor to adult skeletal myocyte, was the first cell type experimented for cardiac regeneration. Skeletal myoblasts were reported to regenerate functional skeletal muscle in response to injury [24, 25]. Owing to the ease of isolation, rapid in vitro expansion, and resistance to ischemia, they have been viewed as a potential candidate to remuscularize the injured heart [26]. Although most of the pre-clinical animal studies [27–32] and clinical studies [33, 34] of skeletal myoblasts have yielded promising results, many of the clinical trials were performed in conjunction with CABG or left ventricular assist device (LVAD) [35, 36], raising the ambiguity of the significant role of skeletal myoblasts in the therapy of heart failure. On the other hand, the therapeutic effects of transplanted BM-MNC in infarcted myocardium showed conflicting outcomes in animal studies. BM-MNC transplanted in a rat model [37] induced angiogenesis at the scar region and improved myocardial function, but no improvement in left ventricle function was observed in sheep [38] and porcine [39] models. The use of bone marrow-derived progenitor cells, such as HSC and EPC have also been studied in patients with both ischemic [40] and non-ischemic [41, 42] cardiomyopathies. The initial clinical results were encouraging but were disputable due to the small sample size. It is therefore unlikely to draw a definitive conclusion on the role of these cells in cardiac repair.

Of all the adult stem cell types, MSCs are in the center of attention for regenerative medicine. These multipotent stem cells were found to be readily available from various tissues and organs [43–48]. Studies have shown that MSCs are able to differentiate into several cell types [49–53] including functional cardiomyocytes in vitro [54, 55] and in vivo [56]. The emerging evidence on the homing potential of MSCs to the site of injury [57–59], coupled with their immunomodulatory properties, has made them more attractive for cardiac tissue repair. Several animal studies have reported the efficient engraftment and evidence of cardiomyogenesis of transplanted MSC at the host injury sites in myocardial infarction (MI) model [60, 61]. For instance, Quevedo et al. demonstrated that in the porcine model, injection of allogeneic male MSCs differentiated into myocyte, vascular muscle, and endothelial cells that integrated into female host myocardium. This was evident by the co-localization of the Y-chromosome with the trilineage markers [62]. Importantly, improvement in cardiac functions after MSC transplantation, as evidenced by the increased left ventricular ejection fraction (LVEF), myocardial blood flow (MBF), and contractility around the infarct border zone, was shown in the study. While extensive clinical trials that exploit MSC to repair the damaged myocardium are currently being conducted, both autologous and allogeneic MSC were generally reported to be safe and effective, except for one trial which showed no beneficial effect when MSCs were delivered through intravenous (IV) injection [63].

### Pluripotent stem cell-derived cardiomyocytes

#### Human ES-derived cardiomyocytes

Unlike adult stem cells, PSCs are highly expandable and can virtually differentiate into any adult somatic cell types, providing an unlimited source of cardiomyocytes. Numerous differentiation techniques have been developed to induce CMs from PSCs [64]. The initial differentiation method involved the embryoid body (EB) formation. EBs are spheroid aggregates that are able to differentiate into cells from all three germ layers within the three-dimensional structures [65]. Kehat and colleagues have demonstrated that human ESC-derived CMs can be isolated from spontaneously differentiated EB. However, this method has been reported to be inefficient and labor-intensive as only approximately 10% of the EBs exhibited spontaneous contractile characteristics and limited amount of CMs could be isolated by mechanical dissection from the beating areas [66]. As such, this EB-based method has then been supplanted by more refined methods that employ defined signaling factors and small molecules to manipulate nodal/activin, BMP4 [67, 68], and Wnt signaling [69, 70], which in turn promote cardiac mesodermal specification.

The feasibility and therapeutic efficacy of hPSC-CMs for cardiac repair after myocardial injury were tested on various animal models. Of note, one encouraging study revealed that the implanted hESC-derived CMs in slow heart-rate guinea pig model facilitated the repair of the infarcted heart and were able to achieve electrical integration with host myocardium [71]. Contradictorily, other researchers reported no significant improvement in cardiac function and limited electrical integration in chronic MI animal models following transplantation [72, 73]. Regardless of these mixed outcomes from the studies, the genuine therapeutic or side effect could be missed or overlooked as there are explicit differences between animal models and human. In order to achieve more precise evaluation of the therapeutic outcome and safety of using PSC-derived CMs in treating cardiac injury, a model with an electrical conduction system that closely resembles that of a human heart is therefore desirable. Accordingly, non-human primate models have been brought into play in various pre-clinical studies. Demonstrated by Chong et al., intramyocardial injection of hESC-CMs improved remuscularization of the infarcted heart in monkeys after ischemic reperfusion (I/R) injury, but complications associated with transient arrhythmia (abnormal heart rhythm) was observed in the study [13]. Another non-human primate MI model study, on the other hand, showed that transplanted human ESC-CMs improved LVEF, with only a subset of the animals experienced graft-associated arrhythmias [74]. The discrepancy in the risk of cardiac arrhythmias in the aforementioned studies was likely attributable to the difference in the disease model (I/R vs. MI), timing of cell delivery, the type, and dose of cardiomyocyte transplanted.

#### iPSC-derived cardiomyocyte

Since ESCs are derived from the inner cell mass of the blastocyst, its usage in research is therefore ethically controversial [75]. The discovery of induced pluripotent stem cell (iPSC) technology, a method of reprogramming somatic cells into pluripotent state using a cocktail of transcription factors, greatly opened up the new dimension of personalized medicine [76], as the use of patient-specific iPSCs to generate autologous grafts could circumvent the possible immune rejection and the ethical issue raised in human ESC. Accumulating evidence has shown that iPSCs display similar characteristics (surface markers, morphology, and growth properties) to that of ESCs [77, 78]. This has spurred interest in investigating the cardiogenic potential of iPSCs [79, 80]. Indeed, iPSC-derived CMs exhibit functional properties of cardiac cells, such as spontaneous beating, contractility, and ion channel expression, suggesting that they can be regarded as a substitution to CMs derived from ESCs in restoring the damaged myocardium [81]. Kawamura et al. have reported that transplantation of hiPSC-derived cardiomyocytes significantly improved LVEF and attenuated left ventricular remodeling in porcine MI model [82]. Whilst tremendous progress has been made in this field, the expectations of iPSC- and ESC-derived CMs have not been met and are yet to be ready for the application of cell therapy in heart failure.

## Translational challenges of PSC-CM in cell-based therapy

### Immature phenotype

Despite the remarkable improvement in developing well-defined differentiation protocols to generate PSC-CM, the maturation status of these cells differs tremendously from that of the adult CMs. Of note, PSC-CMs display immature phenotypes that closely resemble fetal CMs. In terms of morphology, hPSC-CMs appeared to be round and mononucleated, in contrast to adult cardiomyocytes that were binucleated and rod-like in shape [83–85]. In addition, the contractile unit of sarcomeres in hPSC-CMs is shorter and less organized as compared to their mature counterpart. The immaturity of hPSC-CM was also reflected by the absence of transverse tubules (T-tubules) [86, 87], inefficient contractility, difference in metabolism, and electrophysiological properties [88]. On top of the potential risk of arrhythmias upon transplantation, the lack of functional maturation in hPSC-CMs (as described in Table 2) results in inaccurate recapitulation of electrophysiological responses, thereby impeding its translation into clinical application.

**Table 2:** A comparison between human PSC-derived fetal and adult cardiomyocytes

	Characteristics	PSC-CM	Adult CM	References
Morphology	Cell shape	Circular	Rod-shaped	[89–91]
	Nucleation	Single nucleated	25–30% binucleated	[92, 93]
	Mitochondrial content	Slender and long, lesser than in adult CM	Elongated shape, 35% of total cell volume	[94, 95]
	Surface area	1000-1300 μm^2^	10,000-14,000 μm^2^	[96, 97]
Metabolism	Substrate preference	Glucose	Fatty acid	[98, 99]
Sarcomere	Myofibrillar isoform switch (myosin heavy chain)	*β* = α	*β* > > *α*	[100–102]
	Myofibril	Low density	High density	[83, 91, 103]
	Alignment	Random	Anisotropic	[91, 104]
Electrophysiological properties	Upstroke velocity	Slower	Faster	[83, 97]
	Contraction	Asynchronous	Synchronous	[98, 105]

With regard to this, many approaches that aim to enhance the maturation of hPSC-CMs in vitro have been sought and developed [106–109]. Prolonged culture [110], electrical stimulation [111–113], metabolic hormone [114–116], and ascorbic acid (AA) treatments [117] have been shown to induce a more mature phenotype of CMs with more organized sarcomere, improved contractile properties, and a shift in metabolism from anaerobic glycolysis towards oxidative phosphorylation [118]. Strategies including three-dimensional (3D) culture system that co-culture non-CMs and extracellular matrix (ECM) components [108], mechanical force imposed by cyclic stretch [119, 120], as well as microRNAs such as let-7 family, miR-499 and miR-1 [121, 122], were also employed to enhance the maturation process. Despite the emergence of these improved maturation protocols, a standard method to accurately evaluate the level of the maturation of PSC-derived CMs is yet to be defined. Recently, a group of researchers had identified a set of genes with identical relative expression orderings (REOs) within adult cardiac tissue but reversely identical in ESCs [123]. The authors utilized this list of genes to calculate the maturity score and measured the tendency of PSC-CM maturation by comparing the score to that of adult cardiomyocytes. Using this scoring system, they found that the maturity scores of PSC-CMs from 4 different culture methods were on the rise with the extension of culture time (up to 120 days) but were still not reaching the score of adult CM (0.7638 vs 0.9997), suggesting that there is still a gap between mature-like PSC-CMs and adult cardiomyocytes in the heart.

### Diverse cardiomyocyte subtypes (atrial, ventricular, and pacemaker cells)

On top of the variable maturation status in hPSC-CMs, currently available differentiation protocols also generated heterogeneous cell populations that contained atrial, ventricular, and pacemaker cells [124–126]. Many have disregarded the importance of purifying specific cardiac subtypes for subsequent clinical testing, but transplantation of a heterogeneous pool of CM into an infarcted heart might affect the therapeutic outcomes. A detailed review reported that atrial, ventricular, and pacemaker cells possess different cardiac action potential (AP) due to the different roles they play in maintaining cardiac function [127]. The maximal upstroke velocity (Vmax) of ventricular cells is the highest (200–300 V/s), followed by atrial cells (200 V/s) and pacemaker cells (4–5 V/s). Another feature to distinguish different cardiac subtypes is the presence of spontaneous depolarization during phase 4 of the AP in nodal cells. This spontaneous activity is extremely low in atrial cells and is completely absent in ventricular cells [127]. Hence, transplanting multiple subtypes of cardiac cells into the injured heart might lead to arrhythmias as they may not synchronize with the cardiac contractility in the host tissues.

In order to effectively treat the diseases that affect the specific regions of the heart, for example, to remuscularize the ventricular wall of the patient suffering from MI, the ideal approach would be to transplant the population of cells exclusively comprised of ventricular cardiomyocytes. Thus, various sorting and enrichment methods were developed to purify the chamber-specific cardiomyocytes from in vitro differentiated hPSC-CM. Zhang et al. (2011) demonstrated that the addition of retinoic acid (RA) to RALDH2+ mesoderm at the early stage of differentiation induced atrial-like cardiomyocytes at the expense of ventricular cells [128]. Contrarily, inhibition of canonical Wnt pathway by treatment with IWR-1 induced high yield of ventricular cardiomyocytes from hESC-derived cardiovascular progenitor cells [129]. Even with these improvements in chamber-specific cell purification, we are still far from generating a pure population of desired cardiac cell subtypes.

On the other hand, a stable transgenic hPSC line harboring fluorescent reporter under the transcriptional control of human myosin light chain-2V promoter (MLC2V) [130–132] and chick ovalbumin upstream promoter transcription factor II (COUP-TFII) [133] were developed to isolate ventricular and atrial cardiomyocytes, respectively. Despite the robust and high efficiency in enriching specific subtypes after cardiac differentiation of PSCs, the use of virus-based vector, again, has raised safety issues including immunogenicity and insertional mutagenesis risk that hinder their application in future clinical treatment. Among the handful publications about cell-type enrichment, none of the studies reported whether these cells bear resemblance to the right or left chambers of the heart. This could in turn represent another hitch as it was previously reported that the left and right ventricular cardiomyocytes are physiologically different and therefore might affect the outcome of cell therapy [134–136]. For example, owing to their responsibility to pump oxygenated blood to all parts of the body, left ventricular cardiomyocytes are particularly desired in the case of treating MI.

### Low engraftment rate of transplanted cells

The poor survival and engraftment rate of transplanted cells are some of the major unresolved problems that constrain the efficacy of stem cell therapy in treating human diseases. This is due to the fact that transplanted cells are not capable of withstanding the ischemic environment at the site of the injury and the subsequent triggered immune response. Apart from that, the route of administration also affects the engraftment efficiency of hPSC-CM in the heart. Cells can be delivered to the injured myocardium through intramyocardial (IM), intracoronary (IC), or intravenous (IV) injections. Direct IM injection is the most common route of cell administration, but a substantial amount of cell leakage from the punctured holes after injection was reported. Besides, IM, performed through open-chest surgery, is also subjected to high-wall shear stress and acute inflammation [137]. In contrast, injection of cells into the coronary artery or the cardiac vein (IC injection) are mechanical less stressful than IM injection and showed minimal risk of injection-induced ventricular arrhythmias [138]. IV injection, on the other hand, appeared to be the least invasive mode of delivery but with extremely low cell retention as most of the transplanted cells were lost during their travel in the bloodstream and ended up being trapped in the lung, kidney, liver, and other organs [139–141]. Regardless of the route of delivery, engraftment of the transplanted cells to the infarcted myocardium is rare (< 2%) [142, 143].

Several strategies to improve cell survival and engraftment are currently under investigation. In line with the advancement of biomaterial technology, hydrogel has emerged as one of the promising candidates for cardiac tissue engineering. This hydrophilic polymer could provide 3D structural support to the transplanted cell and enhance cell retention in the injured myocardium [144–146]. Using a rat acute MI model, Christman et al. validated the effect of fibrin gel in improving the survival of transplanted cells and consequently the cardiac function. The infarct scar size of the rat injected with myoblast-embedded in fibrin was significantly decreased after 5 weeks post transplantation as compared to the control group (myoblasts embedded with bovine serum albumin). Additionally, the use of fibrin promoted angiogenesis and arteriogenesis in the ischemic myocardium [145]. Other biomaterials, both natural (e.g., collagen, alginate) and synthetic (e.g., peptide nanofibers) were also shown to increase cell survival and stimulate tissue regeneration [147–149].

More recently, tissue engineering technology has also been widely explored for its applicability in cell-based tissue regeneration therapy. The creation of scaffold-free cell sheets, 3D-printed cardiac tissue with extracellular matrix (ECM) and human engineered heart tissue (hEHT), which can be implanted directly to the damaged myocardium has shed light in offering potential solution to the low cell viability and engraftment seen in cell suspension delivery methods [150, 151]. Cell sheet is engineered by culturing the cells on a thermo-responsive polymer surface in monolayer or stacked into multilayers in order to preserved cell-to-cell connection and fabricated 3D cardiac tissues, followed by implantation into the damaged tissue. Transplantation of hiPSC-CM sheets at the myocardial infarct region in a porcine model of ischemic cardiomyopathy improved cardiac performance by structural and electromechanical integration into the host myocardium [82]. Another successful example of cell sheet technology in cardiac regeneration was demonstrated by Sekine et al., in which the author reported that cell sheet transplantation yielded greater cell survival over cell injection, with significant improvement in cardiac function and increased vascularization at the infarcted zone [152]. Using a multiphoton-excited 3D printing technology, hiPSC-derived cardiac muscle patch (hCMP) consisting of hiPSC-CM and other cardiac cell types (hiPSC-EC and hiPCS-SMC) was also generated and evaluated in a mouse MI model. The animals showed improved cardiac functions, reduced infarct expansion, increased vascular density, and cell proliferation [153]. Additionally, transplanted hEHT derived from iPSC-CMs and human endothelial cells (hEC) also resulted in remuscularization of the scar heart tissue and promoted cardiac contractile function of guinea pig model [154]. While moderate success of engineered cardiac-like tissue has been achieved in myocardial repair of animal models, several problems arise. These include (i) short survival period of the transplanted tissue, (ii) ineffective integration to the host tissue resulting from insufficient vascularization, (iii) the need for open-chest surgery, (iv) risk of arrhythmia associated with large graft size, and (v) biodegradability of biomaterials used, all of which collectively hamper the success in clinical application.

### Teratoma formation and immune rejection

Other concerns related to the clinical application of hPSC-derived cells include the risk of teratoma formation and immune rejection following transplantation. The presence of the residual undifferentiated hPSCs, resulting from inefficient differentiation of hPSCs and subsequent poor purification of terminally differentiated cell types, will potentially lead to tumorigenesis. Presently, there is no evidence of teratoma formation after transplantation of hPSC-derived CM into animal models [13, 71, 73, 155–157]. However, more in-depth studies that involve non-primate human models, generation of highly purified mature cardiomyocytes, and long-term follow-up post transplantation are certainly necessary to reassess the safety and efficacy of PSC-derived cell therapy. Moreover, karyotype instability caused by prolonged in vitro culture of PSCs remains a concern for potential neoplastic transformation [158, 159].

Given that most of the PSCs studies employed immunodeficient or immunosuppressed animals [13, 68, 71, 160], the possibility of PSC-based therapies in provoking unwanted immune reaction in clinical testing should not be disregarded. Whilst immunosuppressive therapy post transplantation could improve graft survival rate, undesirable complications such as increased risks associated with infection and malignancy are yet to be resolved [161]. Alternatively, the establishment of diverse human leukocyte antigen (HLA) typed PSC line has also been suggested to expand the number of target patients that can benefit from PSC-based cardiac therapy [162, 163].

## Alternative strategies for successful cardiac regeneration

The concept of cardiac regeneration clings to the idea of repairing and/or replacing damaged cardiomyocytes. It was initially thought that the physical close proximity of PSC-CMs and their subsequent engraftment into the host tissue contributed to the functional recovery of MI in animal models. However, it has recently been suggested that the therapeutic efficacy of these transplanted cells could be executed by paracrine effects. This hypothesis stemmed from the findings that showed functional restoration of the injured murine myocardium despite suboptimal cell engraftment. Furthermore, the observation of abundant secretion of cardio-protective factors involved in anti-apoptotic, pro-angiogenic, pro-cell proliferation, and migration from PSC-CM to the host myocardium implied that the paracrine effects, rather than cell incorporation, could be accountable for the significant therapeutic impact seen in the in vivo studies [164, 165]. Hence, this points towards the development of cell-free therapy that aims to stimulate the endogenous mechanism of regeneration in the heart (Fig. 1). Several innovative strategies have been proposed: (i) administration of cardiac-protective growth factors, (ii) overexpression of therapeutic proteins using modified RNA (modRNA), and (iii) delivery of extracellular vesicles harboring cardiac-regenerative biomolecules. A summary of these strategies are provided in Table 3.

**Table 3:** Summary of cell-free approaches used in cardiac repair

Alternative strategies		Model	Outcome	Type of disease	References
Exosomes	hESC-derived MSCs	Mouse	Reduced infarct size	Myocardial infarction/ reperfusion injury	[166–168]
	hESCSC-derived cardiovascular progenitor	Mouse	Reduced left ventricular end-systolic and end-diastolic volumes	Chronic heart failure	[169]
	hiPSC-derived cardiovascular progenitor		Improved cardiac function through decreased left ventricular volumes and increased LVEF	Myocardial infarction	[170]
	MSCs	Rat	Reduced apoptosis and the myocardial infarct size	Reperfusion injury	[171]
Modified mRNA	Vascular endothelial growth factor (VEGF)-A	Mouse	Induced vascular regeneration	Myocardial infarction	[172]
		Pig	Improved LVEF, increased angiogenesis, and reduced fibrosis		[173]
		Mouse	Promotes Isl1+ to endothelial cell fate		[174]
	Insulin-like growth factors (IGFs)	Mouse	Promote cardiomyocyte survival and abrogate cell apoptosis post-MI		[175]
Growth factors	VEGF	Pig	Increased myocardial blood flow and improved regional ventricular function	Chronic myocardial ischemia	[176]
	Fibroblast growth factors (FGFs)	Mouse	Induced cardiomyocyte proliferation and division	Ischemic heart disease	[177]
	Neuregulin 1 (NRG-1)	Mouse	Induced cardiomyocyte proliferation and promotes myocardial regeneration	Myocardial infarction	[178]
	Periostin	Rat	Reduced fibrosis and infarct size, and increase angiogenesis	Myocardial infarction	[179]
	Hepatocyte growth factor (HGF)	Rat	Reduced apoptosis of cardiomyocytes and lesion size	Reperfusion injury	[180]
	Platelet-derived growth factor (PDGF)	Rat	Decreased infarct size, decreased cardiomyocyte death, and preserved systolic function	Ischemia/reperfusiomy/myocardial infarction	[181]
	Interleukin (e.g., IL-33, IL-11)	Rat	Reduced cardiomyocyte apoptosis, decreased infarct size and fibrosis, and improved ventricular function	Ischemia/reperfusion	[182]
		Mouse	Reduced fibrosis and increase angiogenesis	Myocardial infarction	[183]

### Growth factors

Direct injection of cardiac-protective growth factors into the infarcted heart is the most straightforward way that offers immediate therapeutic effect and allows control over the administered dose. Therapeutic angiogenesis has been viewed as an effective approach in managing the disease as it could induce neovascularization that improves blood supply at the infarcted tissue. In line with this, most of the pre-clinical studies involving growth factor therapy for heart repair focused on vascular endothelial growth factor-A (VEGF) [184–187] and fibroblast growth factor (FGF) [188–190]. Porcine model of chronic myocardial ischemia treated with VEGF, either by IC delivery or direct epicardial implantation, showed significant improvement in the coronary flow [176]. Suppression of cardiomyocyte apoptosis is another strategy to alleviate ischemic injury in the cardiac tissue. Treatment with hepatocyte growth factor (HGF), an anti-apoptotic factor, reduced apoptosis of cardiomyocytes and lesion size in rats [180]. Other anti-apoptotic factors such as platelet-derived growth factor (PDGF) [181] and interleukin (IL) [182, 183] have also been examined for their therapeutic potential in endogenous cardiac repair. Other potential alternatives to these approaches include induction of CM proliferation with acidic fibroblast growth factor (FGF-1) [177], neuregulin (NRG-1) [178], and periostin [179]. The aforementioned growth factors have demonstrated the safety and therapeutic efficacy in treating ischemic heart disease (IHD), particularly MI [191, 192]. However, the issues of short biological half-life, low specificity, and the need of repetitive injection to sustain therapeutic efficacy hamper its progress into clinical application.

### Modified mRNA

On the other hand, endogenous cardiac repair can be achieved through the administration of messenger RNA (mRNA) encoding the protein of interest. Direct transfection of mRNA to the target cells enhances the translation of the desired therapeutic protein, but held back by low stability of mRNA and possible activation of the immune response. Several groups have shown that structural modification of RNA through the incorporation of 5-methylcytidine (5mC), pseudouridine (Ψ), 5-methyluridine (5mU), or N6-methyladenosine repressed Toll-like receptor (TLR) activation, thereby avoid triggering unwanted cellular immune response [193, 194]. Robust but transient expression of the encoded protein induced by modRNA could eradicate the risk of malignancy that would probably be caused by the prolonged expression of the delivered therapeutic molecules, a side effect sometimes seen in conventional gene therapy [195]. A recent noteworthy study revealed that modRNA encoding human VEGF-A injected into the mice infarcted heart resulted in reduced infarct size, ameliorated myocardial perfusion, and improved survival [172]. Importantly, the authors uncovered the underlying mechanism, in which VEGF-A modRNA stimulated the proliferation of epicardial progenitors, followed by its migration to the injured myocardium and subsequent differentiation towards cardiovascular lineage. More recently, modRNAs encoding for IGF1, EGF, HGF, TGFb1, TGFb2, SDF-1, FGF-1, GH, and SCF have been widely investigated in cardiac cells and tissue [172, 174, 175, 196–198]. While most of these modRNAs showed certain level of therapeutic effects, more studies that aim to enhance transfection efficiency of the modRNA and the subsequent translation are fundamental in realizing their application in clinical therapy.

### Stem cell-derived exosomes as cell-free therapy in treating cardiovascular diseases

The discovery of extracellular vesicle, in particular, exosome, as an important messenger in facilitating intercellular communication through transporting and transferring bioactive molecules (proteins and RNAs) between cells has opened a new perspective of applying cell-free therapy in cardiac regeneration [199]. Mouse ESC-derived exosomes have been shown to promote cardiac repair and preserve cardiac function in mouse MI model [200, 201]. In order to enhance the therapeutic effect of exosome in restoring the damaged heart, PSCs could also be genetically modified to secrete exosomes that are enriched with cardio-protective miRNAs, such as miR-21 and miR-210 [202–205]. Indeed, the proof-of-concept of exosome in delivering small RNA molecules to the targeted tissue has recently been confirmed by Alvarez-Erviti et al. [206]. More details about the role of exosome in cardiac regeneration were previously reviewed by Ma et al. [207]. Whilst PSC-derived exosomes offer an innovative approach in stimulating endogenous heart repair, the exact content in exosomes remains elusive. Such unidentified endogenous biomolecules in the exosomes might be transported to the recipient cells and lead to unknown side effects. An in-depth and rigorous characterization of its contents is therefore essential to ensure the safety of applying exosomes in cell-free therapy.

## Conclusion

The therapeutic potential of PSC-derived CM in cell-based therapy in terms of regenerating the damaged heart is evident by various successes in animal models. However, this seemingly promising therapeutic outcomes of PSC-derived CMs should not have overshined the unaddressed issues, particularly the immature phenotype and diverse cardiac cell subtypes that were generated during the differentiation from PSCs. Although many studies have been performed to develop defined differentiation and purification protocols for CMs, it is still unclear to which extent the maturation of PSC-CMs would be ideal for optimal results. Other clinical hurdles, such as tumorigenicity, immunogenicity, and risk of arrhythmias, still remain to be resolved. We are well informed with the favorable and unfavorable impacts of PSC-CMs in animal models, but we know incredibly little about its implication in human. Further studies using humanized animal models are crucial in order to ensure precise prediction of human physiological responses and safety in clinical application. In addition, large-scale production of clinically compliant PSC-CMs is also required for translation into therapeutic reality. As such, stem cell-derived exosome that could possibly regenerate the damaged heart without the involvement of cells appears to be a potential approach to overcome the aforementioned problems of cell-based therapy. To achieve maximal therapeutic outcomes, factors (summarized in Table 4) may be considered in order to generate combinatorial therapies customizable to the severity and the types of heart disease. For instance, PSC-derived cardiac patch would be useful in replacing a large region of ventricular scar, whereas endogenous cardiac regeneration induced by MSC-derived exosome cargo with cardio-protective factors could prevent disease progression.

**Table 4:** Comparison between cell-based and cell-free approaches for cardiac repair and regeneration

	Cell-based therapy			Cell-free therapy		
	Adult stem cells	Embryonic stem cells	Induced pluripotent stem cells	Growth factors	Modified mRNA	Exosomes
Origin	Accessible in various organs and tissues	Derived from embryos	Derived from adult somatic cells	–	–	Cell source dependent
Ethical issues	No	Yes	No	No		
Genetics stability	Genetically stable	Genetically unstable		–		
Safety	No evidence for tumorigenesis	Possible tumorigenic risk		Non-tumorigenic		
Immunogenicity	Low risk of immune rejection	Possible risk of immune rejection	Possible risk of immune rejection (unless autologous)	Non-immunogenic	Non-immunogenic	Depend on the nature of donor cells
Risk of arrhythmia	Low risk of arrhythmia	Possible risk of arrhythmia		No evidence for risk of arrhythmia		
Factors determining therapeutic efficacy	Highly dependent on the state of maturation, cell number, and viability during transplantation			Loading dosage	Protein expression efficiency	Content (biomolecules) in the exosomes
Approach-related limitations	Large number of cells are required for significant therapeutic effect			Short biological half-life and low specificity	May require multiple injections due to transient protein expression	Risk of unknown side effects due to unidentified biomolecules in the exosomes

## Data Availability

Not applicable

## Abbreviations

PSC-CM: Pluripotent stem cell-derived cardiomyocytes
PCI: Percutaneous coronary intervention
CABG: Coronary artery bypass grafting
BM-MNC: Bone marrow mononuclear cells
HSC: Hematopoietic stem cells
EPC: Endothelial progenitor cells
MSC: Mesenchymal stem cells
LVAD: Left ventricular assist device
LVEF: Left ventricular ejection fraction
MBF: Myocardial blood flow
IV: Intravenous injection
EB: Embryoid body
IR: Ischemia reperfusion
iPSCs: Induced pluripotent stem cells
AA: Ascorbic acid
ECM: Extracellular matrix
REOs: Relative expression orderings
AP: Action potential
Vmax: Maximal upstroke velocity
RA: Retinoic acid
MI: Myocardial infarction
MLC2V: Myosin light chain-2V promoter
COUP-TFII: Chick ovalbumin upstream promoter transcription factor II
IM: Intramyocardial
IC: Intracoronary
hEHT: Human engineered heart tissue
hCMP: hiPSC-derived cardiac muscle patch
HLA: Human leukocyte antigen
modRNAs: Modified RNA
VEGF: Vascular endothelial growth factor-A
FGF: Fibroblast growth factor
HGF: Hepatocyte growth factor
PDGF: Platelet-derived growth factor
IL: Interleukin
FGF-1: Fibroblast growth factor
NRG-1: Neuregulin
IHD: Ischemic heart diseases
mRNA: Messenger RNA
5mC: 5-Methylcytidine
Ψ: Pseudouridine
5mU: 5-Methyluridine
TLRs: Toll-like receptors
